# piRNA associates with immune diseases

**DOI:** 10.1186/s12964-024-01724-5

**Published:** 2024-06-28

**Authors:** Mingye Jiang, Xiaoning Hong, Yunfei Gao, Alvin T. Kho, Kelan G. Tantisira, Jiang Li

**Affiliations:** 1 Clinical Big Data Research Center, The Seventh Affiliated Hospital, Sun Yat-Sen University, Shenzhen, Guangdong China; 2 Department of Otolaryngology, The Seventh Affiliated Hospital, Sun Yat-sen University, Shenzhen, Guangdong China; 3 Channing Division of Network Medicine, Brigham and Women’s Hospital, Harvard Medical School, Boston, MA USA; 4 Computational Health Informatics Program, Boston Children’s Hospital, Boston, MA USA; 5 Department of Pediatrics, Division of Respiratory Medicine, University of California San Diego, La Jolla, CA USA; 6 Shenzhen Key Laboratory of Chinese Medicine Active Substance Screening and Translational Research, Guangdong, Shenzhen, China

**Keywords:** PIWI-interacting RNA, piRNA, Immune disease, Autoimmune disease, Immunodeficiency disease, Infectious disease, Biomarker

## Abstract

PIWI-interacting RNA (piRNA) is the most abundant small non-coding RNA in animal cells, typically 26–31 nucleotides in length and it binds with PIWI proteins, a subfamily of Argonaute proteins. Initially discovered in germ cells, piRNA is well known for its role in silencing transposons and maintaining genome integrity. However, piRNA is also present in somatic cells as well as in extracellular vesicles and exosomes. While piRNA has been extensively studied in various diseases, particular cancer, its function in immune diseases remains unclear. In this review, we summarize current research on piRNA in immune diseases. We first introduce the basic characteristics, biogenesis and functions of piRNA. Then, we review the association of piRNA with different types of immune diseases, including autoimmune diseases, immunodeficiency diseases, infectious diseases, and other immune-related diseases. piRNA is considered a promising biomarker for diseases, highlighting the need for further research into its potential mechanisms in disease pathogenesis.

## Background

PIWI-interacting RNA (piRNA) is the largest group of small non-coding RNAs in animal cells, and they directly interact with PIWI (P-element-induced wimpy testis) proteins and primarily function in silencing transposable elements, regulating gene expression, and defending against viral infection [1]. piRNA was first discovered in the testis of *Drosophila melanogaster* in 2001 and was initially considered as a novel long siRNA that mediates *Stellate* gene silencing and maintains male fertility [2, 3]. Subsequently, piRNAs were identified in various species, including worm [4], zebrafish [5], mouse [6, 7] and human [8]. So far, piRNAs have been found in nearly 44 species [9] and are present not only in germ cells but also in somatic cells [10].

piRNAs are 26–31 nucleotides (nt) in length, whereas microRNAs (miRNAs) and small interfering RNAs (siRNAs) range from 19–25 nt [11]. Unlike miRNAs and siRNAs, piRNAs are processed from single-stranded precursors without the involvement of the Dicer enzyme [3]. Most piRNA precursors originate from genomic loci known as piRNA clusters, which are dispersed throughout the genome and are rarely conserved among species [12]. Typical piRNAs contain a uridine and a monophosphate at the 5’ end, and 2’-O-methylation at the 3’ end [13]. This modification increases the stability of piRNAs, allowing them to survive in circulation and body fluids [14, 15].

Although the primary function of piRNA is to inhibit transposon transcription in germ cells and maintain gamete genome integrity [16–18], increasing evidence shows that piRNAs play an important role in various diseases, such as Alzheimer’s disease [19], multiple sclerosis [20], cancer [21], and cardiovascular disease [19, 22–24]. The immune system is crucial in diseases like cancer, and piRNA has been extensively reviewed in cancer immunology [25–27]. Recent studies have reported significant associations between piRNA and immune diseases. In this article, we review recent research on piRNA in various immune diseases, including autoimmune diseases, immunodeficiency diseases, infectious diseases, and other immune-related diseases, highlighting the potential functions of piRNA in the immune system.

### Characteristics, biogenesis and functions of piRNA

#### piRNA characteristics

piRNA, an abbreviation for PIWI-interacting RNA, is a type of single-stranded, small non-coding RNA that interacts with the PIWI-subfamily of Argonaute proteins to form RNA-protein complexes and perform biological functions in animal cells [7, 8]. The length of piRNA ranges from 20 to 36 nucleotides (nt) across various species, typically clustering around 26–31 nt [8, 28]. Mapping piRNA to genomes reveals their uneven distribution, often accumulating and forming clusters [3, 29, 30]. While most piRNAs originate from transposons [3, 29], they can also arise from flanking genomic sequences [31]. In certain cases, such as in *Drosophila* ovaries, murine testes, and Xenopus eggs, some piRNAs may originate from the 3’ untranslated region (UTR) of genes, which are actively selected to produce piRNAs and fulfill specific functions [11, 32]. piRNAs exhibit a uridine bias at the 5’ end and 2’-O-methylation at the 3’ end [5]. Unlike miRNAs, which show conservation across species, piRNA exhibit limited conservation and remarkable diversity [18].

### piRNA biogenesis

piRNA was first identified in the testes of *Drosophila melanogaster* as a novel “siRNA-like” suppressor of *Stellate* [3]. These suppressors, enriched in the repetitive regions of the genome, were also named repeat-associated small interfering RNAs (rasiRNAs) [33]. These rasiRNAs directly bind with the PIWI protein subfamily of the Argonaute family and are now recognized as PIWI-interacting RNAs (piRNAs) [34, 35].

The PIWI protein is a subfamily of the PAZ-PIWI Domain (PPD) protein family, characterized by endonuclease activity [36]. It is expressed not only in the germline but also in somatic cells [37]. PIWI proteins are evolutionarily conserved and play crucial roles in piRNA biogenesis and function (see Table 1) [38, 39]. The human genome contains an additional PIWI gene, PIWIL3 (HIWI3), whose function in piRNA biogenesis remains unknown, as most models are based on *Drosophila* and mice [40].

**Table 1:** PIWI proteins and their homologs in different species

Drosophila	Mouse	Human	C. elegans	Function in piRNA Biogenesis	Ref.
PIWI	PIWIL1 (MIWI)	PIWIL1 (HIWI)	PRG-1	Load piRNA precursor in *Drosophila* and *C.elegans*. Load pachytene piRNA precursor and participate in the ping-pong cycle in mice.	[18, 41]
Aubergine (Aub)	PIWIL2 (MILI)	PIWIL2 (HILI)	-	Participate in the ping-pong cycle in *Drosophila*. Initiate pachytene piRNA biogenesis, load pre-piRNA and cleave piRNA precursor in mice.	[16, 39]
-	-	PIWIL3 (HIWI3)	-	-	-
Argonaute 3 (Ago3)	PIWIL4 (MIWI2)	PIWIL4 (HIWI2)	-	Participate in the ping-pong cycle in *Drosophila*. Load pre-piRNA and participate in the ping-pong cycle in mice.	[16, 17]

**Fig. 1:**
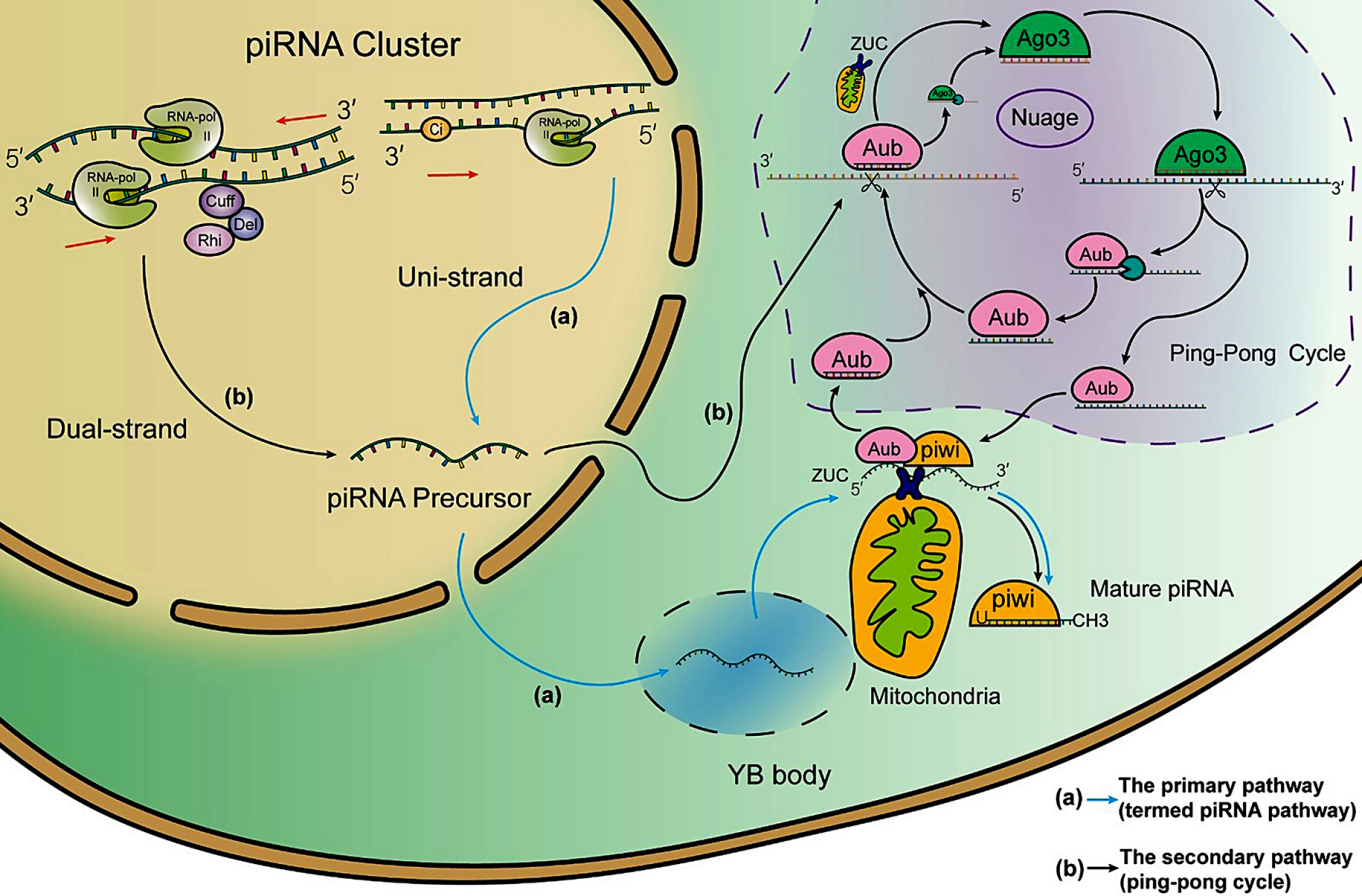
Biogenesis of piRNA. (**a**) Primary piRNA Pathway: The piRNA precursor is generated from a uni-strand piRNA cluster with the transcription factor Cubitus interruptus (Ci) and exported to the Yb body, where the precursor is bound by PIWI protein and cleaved by Zucchini to form pre-piRNA. The pre-piRNA is then trimmed and methylated to become mature piRNA. (**b**) Secondary piRNA Pathway: The piRNA precursor is generated from a dual-strand piRNA cluster with the Rhino–Deadlock–Cutoff (RDC) complex and exported to the nuage, a compartment similar to the Yb body, located near the nucleus. In the nuage, the sense-derived piRNA precursor is targeted by piRNA loaded on Aubergine (Aub) and cleaved into pre-piRNA. This pre-piRNA is loaded onto Argonaut3 (Ago3) and processed into mature Ago3-bound piRNA through the primary piRNA pathway. In turn, the Ago3-bound piRNA then targets the antisense-derived piRNA precursor, generating Aub-bound piRNA. This biogenesis process is known as the ping-pong cycle. *Abbreviations*: Ci: Transcription factor Cubitus interruptus; RDC: Rhino–Deadlock–Cutoff complex

Most piRNAs originate from genomic regions known as piRNA clusters, which span from several to hundreds of kilobases and are notably enriched with repetitive transposable elements (TE) [42]. These piRNA clusters are categorized based on the strand of their original DNA transcription: uni-strand (unidirectional) clusters and dual-stranded clusters (see Fig. 1) [43]. Uni-strand clusters are prevalent in somatic cells, while dual-stranded clusters are predominantly found in germ cells [44]. Recent study have found that Kdm3 can lead to the emergence of de novo piRNA clusters through histone demethylation [45].

piRNA biogenesis involves two primary pathways: the primary (or phased/termed piRNA) pathway and the secondary (or ping-pong cycle) pathway [46]. In *Drosophila* somatic follicle cells, piRNAs are transcribed from uni-strand piRNA clusters by RNA polymerase II and the transcription factor Cubitus interruptus (Ci) [47]. The resulting piRNA precursor undergoes alternative splicing and is exported to Yb body, a cytoplasmic sphere where Yb protein is exclusively localized [48]. Loaded with PIWI protein (PIWI/ Aubergine (Aub)), the piRNA precursor is cleaved into pre-piRNA by Zucchini (Zuc), a putative endonuclease in the outer mitochondrial membrane [49]. After 3’ end trimming and methylation, mature piRNAs are formed. Notably, residual piRNA precursors can be reloaded with PIWI proteins and cleaved again [16]. piRNAs originating from 3’ untranslated regions (3’UTRs) are also generated via the primary piRNA pathway [32]. Additionally, recent studies have identified an endoribonuclease PUCH that can initiate piRNA processing and execute 5′end piRNA precursor cleavage in *C. elegans* [50].

In *Drosophila* germ cells, both the primary and secondary pathways are operational [18, 46, 51, 52]. piRNA precursors from dual-strand piRNA clusters are transcribed by RNA polymerase II and regulated by the Rhino-Deadlock-Cutoff (RDC) nuclear complex, which ensures transcript elongation by suppressing splicing and termination signals (poly(A) signal sequences (PASs)) within the cluster [47, 53]. These precursors are transported to the nuage (meaning ‘cloud’ in French), a perinuclear and membraneless cytoplasmic compartment in the vicinity of mitochondria, where they undergo processing into mature piRNAs through the primary pathway [54]. Furthermore, piRNA precursors from the antisense strand preferentially bind Aub, facilitating their entry into the secondary pathway. Aub-bound mature piRNAs then target and cleave complementary piRNA precursors from the sense strand at the tenth nucleotide from the 5’ end [16, 49, 55]. Subsequently, the cleaved fragments are loaded onto Argonaute3 (Ago3) and processed into mature piRNAs following 3’ end trimming and methylation by Hen1 and Papi [49]. In turn, Ago3-bound piRNAs can target antisense piRNA precursors based on sequence complementarity, initialing further rounds of processing and amplification through the ping-pong cycle [56].

### piRNA functions

#### piRNA and transposon silencing

The primary and ancestral function of piRNAs is to silence transposons, thereby protecting the germline genome from transposon attacks [29, 57, 58]. The PIWI-RNA complex accomplishes this at both transcriptional and post-transcriptional levels, depending on the specific PIWI proteins involved [16].

During transcriptional gene silencing (TGS), PIWI-bound piRNA (termed piRNA) are transported to the nucleus where they bind to nascent transposon transcripts via base-pairing. PIWI proteins then recruit cofactors to inhibit transcription, effectively silencing transposons. In Drosophila, these cofactors include Maelstrom (Mael), Panoramix (Panx, also known as Silencio), Asterix (Arx, also known as Gtsf1), and the SUMO ligase Su(var)2–10 [16]. Mael interacts with the SWI-SNF chromatin remodeling complex, reducing RNA polymerase II occupancy on transposon promoters in somatic cells [59]. Panx forms a complex with Nxf2 and Nxt1 [58], recruiting heterochromatin remodelers such as the H3K9 methyltransferase Eggless (Egg), the H3K4 demethylase Lsd1, and heterochromatin protein 1 (HP1) [60, 61]. Arx is crucial for PIWI to recruit Mael and Panx [59], while Su(var)2–10 links PIWI with Panx and Arx [62, 63].

In post-transcriptional gene silencing (PTGS), the ping-pong cycle is vital for transposon silencing through Aub-bound and Ago3-bound piRNAs [46]. As the ping-pong cycle genertes secondary piRNAs, transposon mRNAs are degraded, leading to transposon silencing [49].

#### piRNA and epigenetic modification

Beyond transposon silencing, piRNAs play roles epigenetic regulation [64]. piRNA can regulate gene expression by methylating CpG islands of target genes through DNMT1 and DNMT3α [65]. The PIWI-RNA complex interacts directly with METTL3, inhibiting mRNA N6-methyladenosine (m6A) modification [66]. piRNAs bind to hnRNPU and interact with ubiquitin-specific protease 8 (USP8) to facilitate the deubiquitination of target proteins [67]. They can also interact with N-acetyltransferase 10 (NAT10) to enhance ac^4^ C acetylation of target mRNA transcripts [68]. The epigenetic functions of piRNAs have been extensively studied in various cancers due to their impact on gene expression [69, 70].

#### piRNA and targeting mRNA

piRNAs can target protein-coding sequences of mRNAs and modulate gene expression [56, 71]. Although the targeting mechanism varies across species, it is believed to be sequence-driven yet not entirely sequence-specific [72]. In C. elegans and mice, the nucleotides at positions 2–8 and 14–22 are crucial for piRNA targeting [73, 74]. Interestingly, random mismatches within the piRNA sequence do not significantly affect its targeting efficiency [75].

Additionally, nuclear piRNAs can target pre-mRNA introns through sequence complementarity, leading to downregulation of gene expression in somatic cells [76]. Alternatively, they recruit splicing factors to enhance pre-mRNA splicing, a function conserved in mouse and *Drosophila* germ cells [77]. Notably, transposon sequences are present not only in the 3’UTR but also in the 5’UTR and coding sequences (CDSs) of mammalian mRNAs [78]. piRNAs can degrade these mRNAs via their transposon sequences, thereby regulating gene expression [75, 79, 80].

#### piRNA and viral defence

piRNAs also play a role in defending against viruses in certain species [81]. In mosquitoes, the ping-pong cycle is utilized to degrade viral RNAs when infected by positive-strand, single-stranded RNA (ssRNA) viruses [58]. However, antiviral defence via piRNA is not observed in *Drosophila*, potentially due to a lower diversity of PIWI proteins and loss of function during evolution [82].

### piRNA database

Multiple databases have been established to comprehensively collect and annotate piRNA data [83]. Databases such as piRBase [9], piRNAdb [84], and piRNABank [85] not only house piRNA sequences but also include information on their biogenesis, functions, target predictions, and disease associations. Other databases, including piRNAclusterDB [12], piRNAQuest [86], piRTarbase [87] and piRDisease [88], focus on specific aspects of piRNA research, such as piRNA clusters, piRNA targets, and the association between piRNAs and diseases. These databases are invaluable resources that significantly advance our understanding of piRNA functions and their roles in diseases (Table 2).

**Table 2:** Commonly used piRNA databases

Database	Description	Ref.	Link
piRBase	piRBase contains more than 181 million unique piRNA sequences covering 440 datasets from 44 species. Most piRNAs are derived from literature and external databases and collected through a variety of experiments, including chromatography, Piwi protein IP, Piwi protein CLIP-seq, etc.	[9]	http://bigdata.ibp.ac.cn/piRBase/index.php
piRNAdb	piRNAdb compiles a catalog of 27,329 piRNAs, 23,380 potential target genes, and 47,060 related gene ontology terms, all of which are systematically organized and interconnected with the respective piRNAs. piRNAs are collected from immunoprecipitation or small RNA-Seq.	[84]	https://www.pirnadb.org/
piRNABank	piRNABank is a very user-friendly resource containing empirically validated sequences and related information for piRNAs that have been reported in human, mouse, and rat.	[85]	http://pirnabank.ibab.ac.in/
piRNAclusterDB	piRNAclusterDB contains more than 350 Sequence Read Archive (SRA) datasets covering 51 different species, totaling more than 15,000 piRNA clusters.	[12]	https://www.smallrnagroup.uni-mainz.de/piRNAclusterDB/
piRTarBase	piRTarBase provides a user-friendly interface to access predicted and experimentally determined piRNA target sites and their regulatory effects on worm endogenous genes.	[87]	http://cosbi6.ee.ncku.edu.tw/piRTarBase/
piRDisease	piRDisease integrates experimentally supported data on the relationship between piRNAs and multiple diseases, providing 7939 manually curated associations of 4796 experimentally supported piRNAs implicated in 28 diseases.	[88]	http://www.piwirna2disease.org/index.php
piRNA-IPdb	piRNAIPdb selects 23 datasets from piRBase and after processing with a custom bioinformatics pipeline, it contains 18,904,992 unique sequences identified after immunoprecipitation with any of the PIWI proteins in mouse.	[89]	https://ipdb2.shinyapps.io/ipdb2/
piRNAQuest	piRNAQuest is a comprehensive piRNA platform that contains 92,77,689 unique piRNAs from 28 species. It characterizes piRNAs based on their genomic location and predicts piRNA clusters and target genes.	[86]	http://dibresources.jcbose.ac.in/zhumur/pirnaquest2/start.php

### piRNA and autoimmune diseases

Autoimmune diseases are immune disorders characterized by an excessive or abnormal response of the immune system to self-antigens, leading to continuous damage to organs and tissues [90]. The incidence rate of autoimmune diseases in the general population can reach 3–5%, with a significantly higher rate among first-degree relatives and monozygotic twins [91].

Both environmental and genetic factors are primary drivers of autoimmune diseases [91]. Environmental triggers, encompassing drugs, chemicals, infectious agents, cigarette smoke, and ultraviolet radiation, can elicit autoimmune responses through diverse mechanisms [92]. Genetic predispositions, including monogenic mutations and complex genetic variations, directly influence the autoimmune response and determine antigen specificity [93]. Notably, the major histocompatibility complex (MHC) locus has been identified as a significant risk factor for autoimmune diseases [94]. Common examples of autoimmune diseases include rheumatoid arthritis, systemic lupus erythematosus, multiple sclerosis, type 1 diabetes, primary biliary cirrhosis, Graves’ disease, and Crohn’s disease [91, 92, 95].

T helper 1 (Th1) and T helper 2 (Th2) CD4^+^ T cells cross-regulate each other, and the Th1/Th2 balance significantly impacts on autoimmune inflammation [96]. Autoimmune diseases caused by a Th1 response are mediated by cytokines from Th1 cells (e.g., IL-2, IFNγ and TNFα) and macrophages (e.g., IL-1, IL-6 and IL-12), whereas those caused by a Th2 response are mediated by cytokines such as IL-4, IL-5 and IL-13 [97].

### piRNA in rheumatoid arthritis

Rheumatoid arthritis (RA) is a chronic autoimmune disease closely related to the immune system [98]. It affects synovial joints and cause systemic inflammation [99]. Synovial fibroblasts (SF) are crucial effector cells in the pathogenesis of RA, as they attach to cartilage and promote joint destruction [100].

Pleštilová et al. identified up to 300 piRNAs through small RNA sequencing in synovial fibroblasts from RA and osteoarthritis (OA) patients [101]. Among these, the top three piRNAs with the highest expression level were piR-16,735, piR-4153 and piR-823, while the piRNA with the lowest expression level was piR-16,659. The expression levels of most piRNAs were positively correlated with each other, implying a potential co-regulated mechanism. piR-4153, piR-16,659 and piR-823 exhibited less tightly regulated patterns in RA, suggesting different regulatory factors between RA and OA. Additionally, the expression of PIWIL2 and PIWIL4 significantly increased when SF were treated with TNFα + IL1β/TLR-ligands, and Poly(I: C) significantly increased the expression of piR-16,659 [101, 102].

Ren et al. identified 1565 known piRNAs in peripheral leukocytes of RA patients, of which 15 piRNAs were upregulated and 9 piRNAs were downregulated. The selected piRNAs were validated by RT-qPCR among 42 RA patients and 81 healthy controls. piR-hsa-27,620 and piR-hsa-27,124, which are immunoregulation-related piRNAs, showed promise as biomarkers for RA with an area under the ROC curve (AUROC) of 0.79 and 0.74, respectively, indicating a good ability to distinguish RA from healthy individuals (AUROC is a performance metric used to evaluate classification models) [103].

### piRNA in systemic lupus erythematosus

Systemic lupus erythematosus (SLE) is a multisystem autoimmune disease that can severely affect women aged 10–50 years [104]. The global prevalence of SLE is 43.7 (ranging from 15.87 to 108.92) per 100,000 persons, with much higher rates among Asians, Hispanics and Africans [105]. The pathogenesis of SLE is complex, involving many risk factors including diseases (e.g., atopic dermatitis), lifestyle choices (e.g., smoking and drinking), and genetic polymorphisms (e.g., genes related to B cell and T cell functions) [106]. SLE can damage tissues and organs in the body, leading to various complications such as lupus nephritis (LN) [107].

Flores-Chova et al. identified five piRNAs that were differentially expressed in exosomes of SLE patients with LN, including piR-020244, piR-013323, piR-009000, piR-020364 and piR-001184. Additionally, they found two piRNAs differentially expressed in SLE patients without LN, namely piR-019949 and piR-018624 [108]. According to the piRNAdb annotation, piR-020244 is associated with the mitochondrial inner membrane and cytochrome-c oxidase activity. However, further information could not be determined due to the lack of comprehensive guidelines on piRNA bioinformatics analysis.

### piRNA in multiple sclerosis

Multiple sclerosis (MS) is a chronic neuroinflammatory disease affecting approximate 2.3 million people worldwide [109]. Both environmental and genetic risk factors contribute to the pathogenesis of MS, with the adaptive immune system, particularly T cells and B cells, playing a crucial role [109, 110].

Kamenova et al. investigated endogenous miRNAs and piRNAs that regulate MS candidate genes using bioinformatic methods [20]. They identified 50 piRNAs from 40,000 piRNAs in the database as potential regulators of 21 MS candidate genes. Seven of these genes, including FCRL3, HLA-DRB1, MAPK1, MLANA, MYC, TALDO1 and TRIP11, were exclusively targeted by piRNA. Some candidate genes could be targeted by multiple piRNAs; for example, MYC could be targeted by piR-16,771, piR-17,232 and piR-1177. This study suggests that piRNA may be involved in MS pathogenesis by regulating these target genes, but further wet-lab validation is needed.

**Fig. 2:**
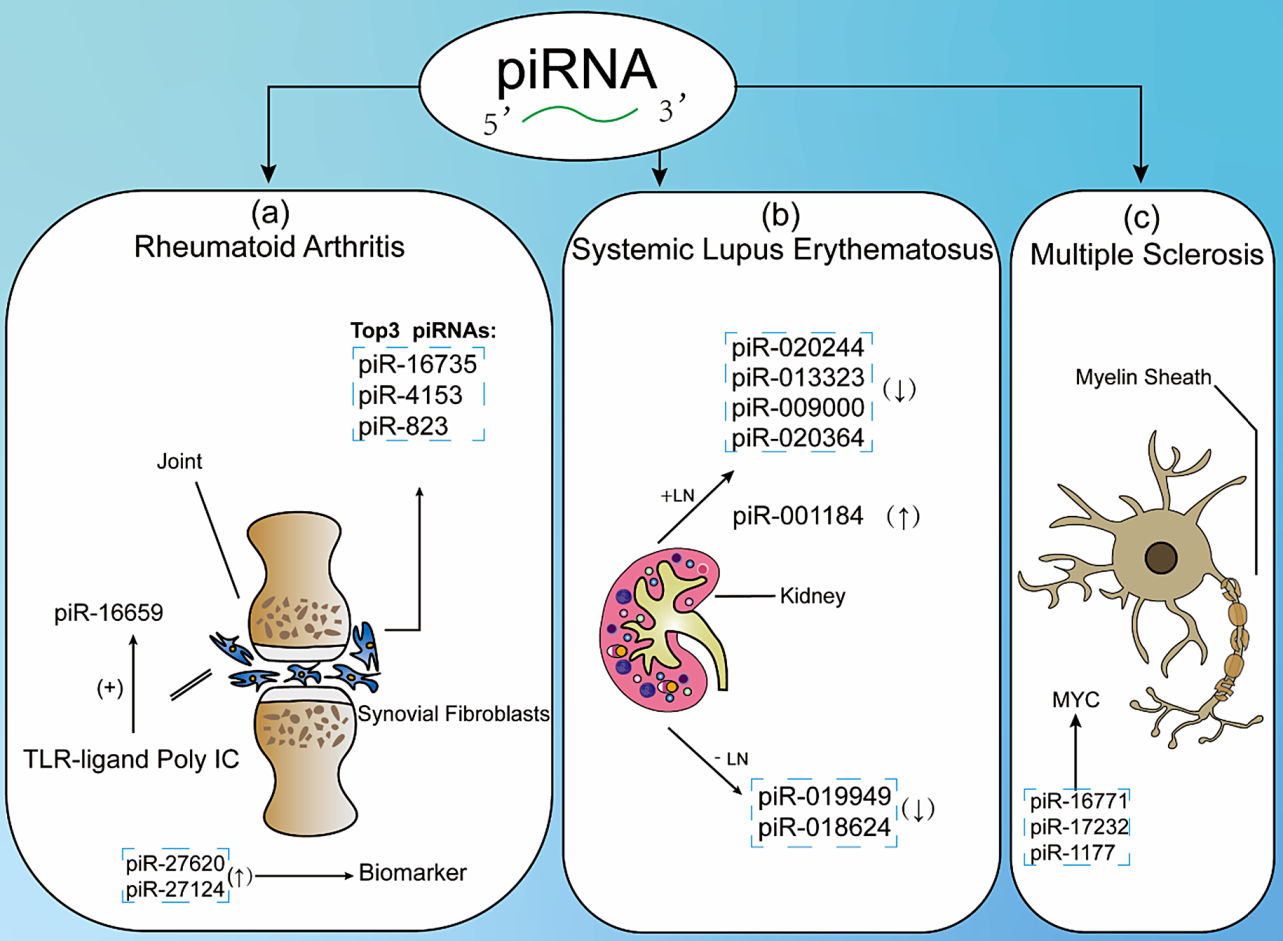
piRNAs in Autoimmune Diseases. (**a**) piR-16,735, piR-4153 and piR-823 are the most expressed piRNAs in synovial fibroblasts among RA patients. TLR-ligand and Poly (I: C) could increase the expression of piR-16,659. piR-27,620 and piR-27,124 are significantly up-regulated in rheumatoid arthritis patients and could serve as potential biomarkers. (**b**) piR-020244, piR-013323, piR-009000, and piR-020364 are upregulated, while piR-001184 is downregulated in systemic SLE patients with LN. piR-019949 and piR-018624 are downregulated in SLE patients without LN. (**c**) piR-16,771, piR-17,232 and piR-1177 can target the MS candidate gene MYC. *Abbreviations*: RA: Rheumatoid Arthritis; SLE: Systemic Lupus Erythematosus; LN: Lupus Nephritis; MS: Multiple Sclerosis

### piRNA and immunodeficiency disease

Immunodeficiency diseases result from the failure or absence of elements of the immune system and can be either primary or secondary [111]. Primary immunodeficiency is caused by the genetic anomalies and can be divided into monogenic immunodeficiency and polygenic immunodeficiency [112]. Secondary immunodeficiency is often caused by extrinsic factors such as virus infection, radiation, severe malnutrition and immunosuppressants, leading to temporary or permanent damage to the immune system [113]. Secondary immunodeficiency is more common than primary immunodeficiency [111]. Early diagnosis of immunodeficiency diseases can help identify the cause, and early intervention can improve the patient’s condition [114].

T cell dysregulation is significant in immunodeficiency diseases, with CD4 + T cells being strongly inversely correlated with the severity of these diseases [115]. Mutations in the common cytokine receptor γ-chain (γc) and defects in cytokine signaling are both drivers of pathology [116].

### piRNA in AIDS

Acquired Immune Deficiency Syndrome (AIDS) is an immunodeficiency disease caused by infection with the Human Immunodeficiency Virus (HIV), leading to a widespread loss of CD4 + T cell [117]. Approximately 37.7 million people worldwide are infected with HIV [118], and patients with HIV have an increased risk of inflammation in tissues and organs [119].

Doke et al. discovered significant alterations in the expression of piRNAs and PIWI proteins in human primary astrocytes exposed to HIV-1 Tat and cocaine [120]. They identified 239 differentially expressed (DE) piRNAs, with functional analysis revealing that most target genes were enriched in critical biological processes, particularly metabolic pathways, gluconeogenesis, and sugar metabolism. This study implies that exposure to HIV-1 Tat and cocaine may disrupt piRNA expression, potentially affecting the nervous system through impaired energy metabolism in astrocytes. Martínez-González et al. identified 39 piRNAs from plasma extracellular vesicles in HIV/HCV coinfected patients, although the role of these piRNAs remained unclear [121]. Yu et al. found that the extracellular vesicles (EVs) isolated from murine hypothalamic neural stem/progenitor cells (htNSC) and hippocampal NSC had stronger antiviral functions when the host cells were treated with HIV-based lentivirus. They examined piRNAs from these EVs and found that two piRNAs were significantly upregulated, suggesting that piRNAs are involved in antiviral actions [122]. Additionally, Peterlin et al. found that the human PIWI protein Piwil 2 (Hili) was expressed in activated CD4 + T cells, where it bound to tRNAs, including some rare tRNAs whose codons are overrepresented in the HIV genome, thereby inhibiting its replication [123].

### piRNA in chronic lymphocytic leukemia

Chronic lymphocytic leukemia (CLL) is one of the most common leukemias in adults, characterized by the malignant proliferation of lymphocytes in the blood, especially CD5 + CD23 + cells in the blood, marrow, and secondary lymphoid tissue [124]. The main symptoms of CLL include swollen lymph nodes, fatigue, petechiae, easy bruising or bleeding, fever, and infection [125]. Besides, antibody generation reduction and abnormal cellular immunity are reported in many CLL patients [126].

Kaur et al. identified two novel sequences homologous to piR-36,225 and piR-30,799 from mononuclear cells in CLL patients. These potential piRNAs had elevated expression levels in CLL, suggesting that piRNAs may be associated with chronic lymphocytic leukemia [127].

### piRNA in diffuse large B-cell lymphoma

Diffuse large B-cell lymphoma (DLBCL) is the most common form of non-Hodgkin’s lymphoma, accounting for approximately one-third of all lymphomas [128]. It is characterized by abnormal, larger B cells that have stopped responding to signals and accumulated in lymph nodes [129]. DLBCL patients primarily present with lymph node disease accompanied by fever, weight loss, drenching night sweats, or other symptoms [130].

Han et al. reported that the piRNA-30,473/WTAP/hexokinase 2 (HK2) axis played an important role in the pathogenesis of DLBCL [131]. They found piRNA-30,473 was elevated in DLBCL patients and associated with an aggressive phenotype and poor prognosis. piRNA-30,473 could mediate m6A methylation by regulating the expression of WTAP, which in turn increased the expression of HK2 and promoted the progression of DLBCL. piR-30,473 may serve as an independent biomarker for DLBCL.

**Fig. 3:**
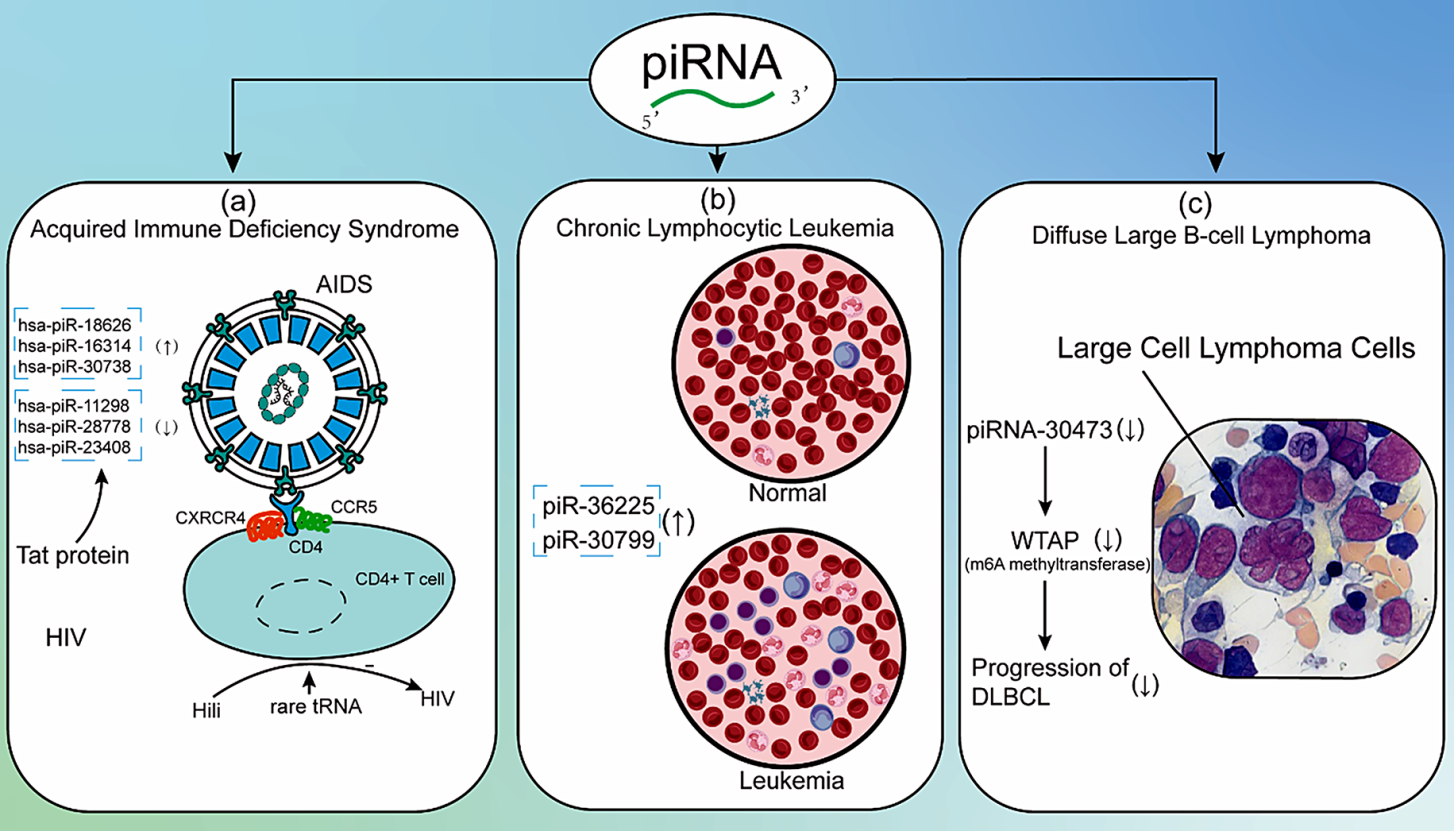
piRNA in Immunodeficiency Disease. (**a**) The expression of piRNA is altered in astrocytes when exposed to HIV Tat (upregulated: hsa-piR-18,626, hsa-piR-16,314 and hsa-piR-30,738; downregulated: hsa-piR-11,298, hsa-piR-28,778 and hsa-piR-23,408). Additionally, Hili can inhibit HIV replication in activated CD4 + T cells by binding to rare tRNAs. (**b**) piR-36,225 and piR-30,799 show elevated expression levels in CLL patients. (**c**) piRNA-30,473 mediates m6A methylation by regulating WTAP expression, which in turn increases HK2 expression and promotes the progression of DLBCL. *Abbreviations*: Hili: Human PIWI Protein; CLL: Chronic Lymphocytic Leukemia; DLBCL: Diffuse Large B-Cell Lymphoma

### piRNA and infectious disease

Host cells defend against pathogens through intrinsic immunity, also known as cell-autonomous immunity [132]. During invasion, pattern recognition receptors (PRRs) detect pathogen-associated molecular patterns and activate antiviral interferon and pro-inflammatory responses [133]. Interferon spreads between cells and activates the expression of interferon-stimulated genes (ISG) through the JAK-STAT signaling pathway. Most ISGs have antiviral effects, such as degrading viral nucleic acids and inhibiting viral gene expression [134].

Eukaryotic genomes harbor sequences originating from viruses, known as endogenous viral elements (EVEs) [135]. Recent studies have revealed that these EVEs are abundant in piRNA clusters across numerous mammalian species, including humans and arthropods [136]. These EVEs undergo transcription and contribute to the biogenesis of piRNA. The piRNA derived from EVEs can be antisense or complementary to ancient viral sequences. In the event of reinfection, these EVE-derived piRNA can direct PIWI proteins to target viral RNAs, effectively silencing them, reminiscent of the CRISPR-Cas immune system found in prokaryotes [137].

The ping-pong cycle in the piRNA biogenesis is also exploited to defend against viral infection in mosquitos, where PIWI5-bound and Ago3-bound piRNA cleave the sense and anti-sense strands of viral RNAs to produce piRNA [138]. However, this function was not observed in *Drosophila*, possibly due to a loss during evolution [82].

### piRNA in viral infection

#### SARS-CoV-2

Ikhlas et al. induced exosomes/microvesicles (Ex/Mv) in murine hypothalamic neural stem cells (htNSCs) using fragments of the SARS-CoV-2 genome. These Ex/Mv contained increased levels of piRNA. Knockout of PIWIL2 decreased piRNA expression in Ex/Mv and reduced antiviral effects in htNSC, implying that the PIWI-piRNA system plays a crucial role in antiviral function and viral immunity [139].

Akimniyazova et al. identified piRNA that could interact with SARS-CoV-2 genome through bioinformatics methods. They identified four clusters of piRNA binding sites in the SARS-CoV-2 genome, comprising 13 piRNAs, 39 piRNAs, 24 piRNAs, and 12 piRNAs. They also proposed synthetic piRNA to inhibit the proliferation of coronaviruses [140].

#### Herpes simplex virus (HSV)

Wang et al. studied the global expression profile of piRNA in HSV-1 infected human fibroblasts [141]. They identified 69 differentially expressed piRNAs (52 upregulated and 17 downregulated), which were enriched into antiviral immunity functions. piRNA transfection assays in HSV-1 infected fibroblasts revealed that piR-36,233 inhibited HSV-1 replication, while piR-36,041 promoted HSV-1 replication, suggesting piRNA involvement in the viral replication process in host cells.

Liu et al. found a strong association between integrated herpesvirus 6 and MOV10L1 from a GWAS of 141,431 Chinese women who attended non-invasive prenatal testing [142]. MOV10L1 is a PIWI-interacting RNA helicase and helps pre-piRNA load onto PIWI proteins [143]. Although this research does not study piRNA directly, it implies an indirect link between piRNA and HSV infection.

#### Respiratory syncytial virus (RSV)

Corsello et al. studied piRNA expression in RSV-infected human small airway epithelial cells [144]. They identified differentially expressed piRNAs at various time points using a piRNA microarray: 548 piRNA (6 h), 897 piRNAs (15 h), and 1644 piRNAs (24 h). 157 piRNAs were differentially expressed among all three time points and the top 14 differentially expressed piRNAs were validated by RT-qPCR. Target gene enrichment analysis indicated significant functions in immunological memory, suggesting piRNA participation in the immune response to pathogens.

#### Rhinovirus

Li et al. examined piRNA expression profiles in human rhinovirus (HRV)-infected H1-HeLa cells by high-throughput sequencing at different time points [145]. They identified 21 differentially expressed piRNAs throughout all the time points and validated them by RT-qPCR. Some downregulated piRNA were positively associated with LINE-1 transcription or retrotransposons’ expression, implying a potential mechanism of piRNA in rhinovirus infection.

#### Human papillomavirus (HPV)

Human papillomavirus infection is an important risk factor for head and neck squamous cell carcinoma (HNSCC) [146]. Firmino and Martinez et al. studied piRNA expression patterns in 498 non-malignant and tumor tissues from HNSCC patients. They found that piRNAs were differentially expressed in HPV-positive and negative cases and that piRNA expression patterns could predict overall survival in HPV-positive HNSCC patients [147].

Krishnan and Qu et al. examined differentially expressed piRNAs between HPV16 (+) HNSCC and HPV(-) normal controls and associated HPV-dysregulated piRNA with PIWI proteins, RTL family genes, HNSCC-associated genomic alterations, and clinical features [148]. Their study highlighted the crucial role of piRNA in HPV-related HNSCC.

#### piRNA in tuberculosis

Tuberculosis (TB) is an infectious disease caused by *Mycobacterium tuberculosis*, affecting not only the lung but also other parts of the body [149]. TB can remain dormant in the host’s body for extended periods of time before becoming active, causing fever, chills, night sweats, weight loss, and fatigue [150]. TB affects a quarter of the world’s population and increases by 1% annually [151]. piRNAs may serve as good biomarkers for TB diagnosis [152–154].

De Araujo et al. identified 35 piRNAs from 8 patients with TB, 21 patients with latent TB infection (LTBI), 6 treated patients with LTBI and 14 healthy controls [155]. Seven piRNAs, including piR_017936, piR_019675, piR_019912, piR_020548, piR_020381, piR_020490 and piR_009059, were differentially expressed (DE) between the LTBI and treated LTBI groups. piR_020381, piR_020490 and piR_009059 were also DE between LTBI and healthy controls. The expression of piR_009059 was significantly lower in LTBI than in the other three groups. piR_017936 differentiated LTBI from healthy controls with an AUROC of 0.72. Eleven piRNAs were DE between TB and the other three groups, of which piR_001421, piR_018570 and piR_020582 were associated with TB globally. piR_017936 was associated with the short-term interferon gamma release assay (IGRA), an in vitro blood test used in the diagnosis of some infectious disease, especially tuberculosis. It could differentiate latent TB infection (LTBI) from uninfected exposed controls (ExC) with an AUROC of 0.72 (*P* = 0.018), suggesting a good biomarker for LTBI.

Zhang et al. investigated the piRNA expression in 20 pulmonary tuberculosis patients and 20 healthy controls through peripheral blood samples [156]. They identified 777 DE piRNAs, with 192 exclusively expressed in TB patients and 142 exclusively expressed in healthy controls. They predicted piRNA target genes using Miranda [157] and performed GO and KEGG enrichment analysis for DE piRNAs, showing most enriched pathways related to immunity.

### piRNA in bacterial infection

#### Mycobacterium leprae

Leprosy is an infectious disease caused by the Mycobacterium leprae, leading to chronic granulomatous infection in the skin and peripheral nerve [158]. Pinto et al. identified 14 differentially expressed piRNAs between leprosy patients and healthy controls, with 5 piRNAs (piR-hsa-12,454, piR-hsa-1580, piR-hsa-21,131, piR-hsa-27,007 and piR-hsa-28,634) showing excellent predictive ability for leprosy with AUROC greater than 0.9. The target genes of piRNA, predicted by miRanda [157], were analyzed for enrichment, revealing that NF-kappaB signaling and epithelial cell development are significant biological processes in leprosy infection. The target genes GAS6 and IL6R may contribute to nerve regeneration [159].

#### Brucella

Brucellosis is a zoonosis caused by Brucella bacteria, infecting goats, cattle, pigs, and other animals [160]. People may contract the infection through unpasteurized milk and other secretions from infected animals, resulting in fever, sweating, weakness, arthritis, and lymphadenopathy [160]. Wang et al. identified 7 upregulated piRNAs in brucellosis patients and validated them through qRT-PCR. Three piRNAs, including piR-000753, piR-001312 and piR-016742, could be potential biomarkers for brucellosis, with AUROC ranging from 0.7 to 0.8 [161].

**Fig. 4:**
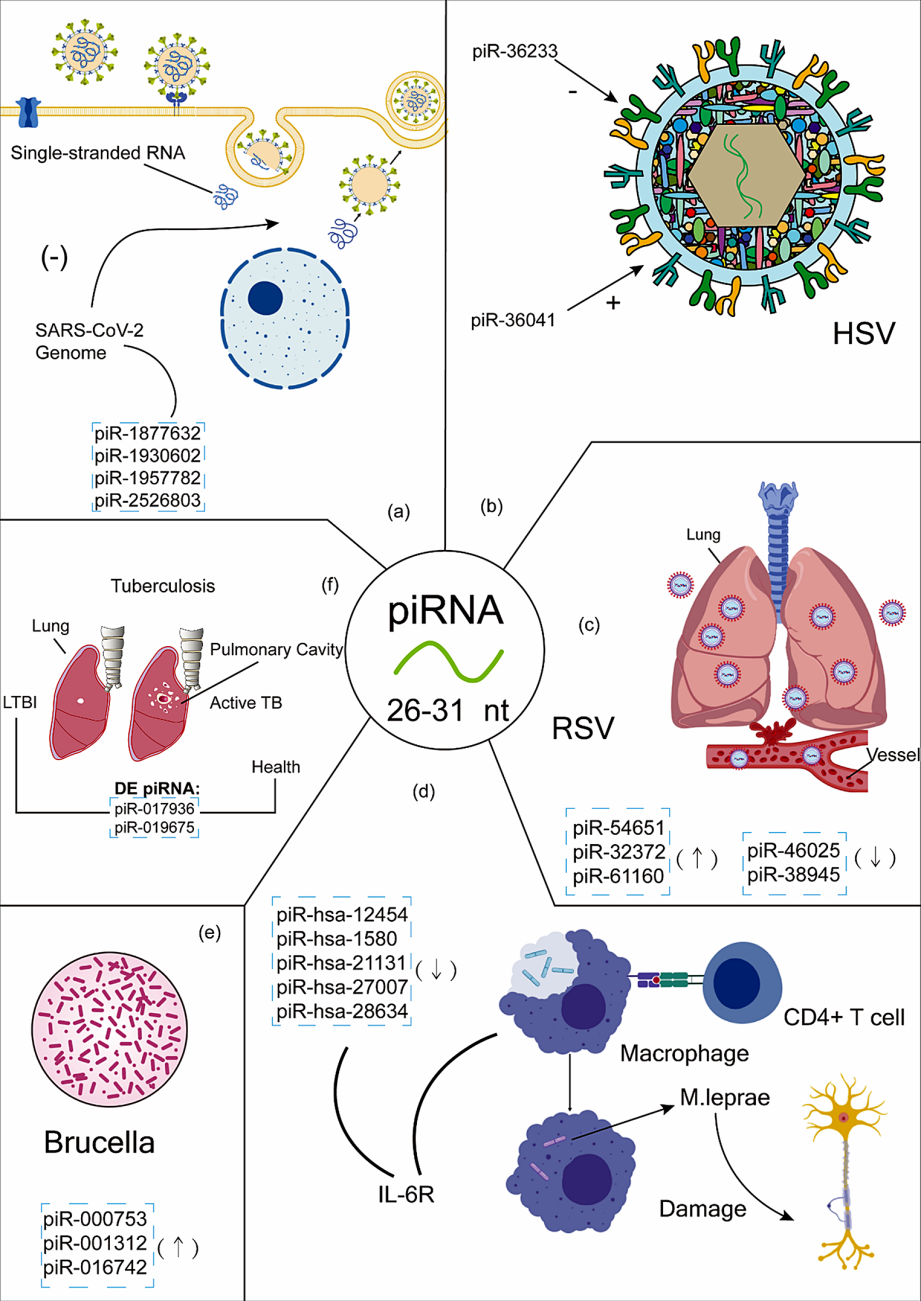
piRNA in Infectious Disease. (**a**) In silico analysis shows piR-1,877,632, piR-1,930,602, piR-1,957,782 and piR-2,526,803 can interact with the SARS-CoV-2 genome and inhibit its replication. (**b**) piR-36,233 inhibits HSV-1 replication, while piR-36,041 promotes it. (**c**) piR-54,651, piR-32,372, piR-61,160, piR-46,025 and piR-38,945 are differentially expressed in RSV-infected human small airway epithelia cells. (**d**) piR-hsa-12,454, piR-hsa-1580, piR-hsa-21,131, piR-hsa-27,007 and piR-hsa-28,634 are significantly differentially expressed in leprosy patients. The predicted target genes, IL-6R and GAS6, are involved in neuroregeneration. Downregulation of piRNA targeting IL-6R can promote its expression on macrophage membrane. (**e**) piR-000753, piR-001312 and piR-016742 are significantly upregulated in brucellosis patients and could serve as good biomarkers. (**f**) piR-017936 and piR-019675 are potential biomarkers for LTBI. *Abbreviations*: SARS-CoV-2: Severe Acute Respiratory Syndrome Coronavirus 2; HSV: Herpes Simplex Virus; RSV: Respiratory Syncytial Virus; M. leprae: Mycobacterium Leprae; LTBI: Latent Tuberculosis Infection

### piRNA and other immune-related diseases

#### piRNA in asthma

Asthma is a heterogeneous disease characterized by airway inflammation, leading to mucus production, hyperresponsiveness and airway remodelling [162]. It may affect 300 million people worldwide, with higher incidence and prevalence in children compared to adults [163]. Based on the relative content of T helper 2 (Th2) cells, asthma is commonly divided into two major endotypes: Th2-high and Th2-low endotypes [164, 165].

Alexandrova et al. identified five differentially expressed piRNAs (piR-34,456, piR-35,550, piR-61,298, piR-32,376 and piR-35,413) in bronchial smooth muscle (BSM) cells between asthmatic patients and healthy controls, although their roles in asthma pathogenesis remain unknown [166]. Zhong et al. discovered that piR-30,840 can downregulate interleukin-4 (IL-4) expression by binding to the pre-mRNA intron region, thereby regulating the development of Th2 lymphocytes through IL-4 [76].

Li et al. identified 15 piRNAs associated with eosinophil count and 11 piRNAs associated with serum total IgE in the Childhood Asthma Management Program (CAMP), a randomized clinical trial for evaluation of ICS treatment. Of these, three piRNAs (piR-33,520, piR-35,174 and piR-33,064) and one piRNA (piR-43,770) were replicated in another cohort, the Genetics of Asthma in Costa Rica Study (GACRS). Mediation analysis showed that piRNAs may affect long-term asthma exacerbation through eosinophils and IgE. Additionally, piRNAs were also found to be excellent biomarkers for T2-high asthma, with AUROC values ranging from 0.91 to 0.95 [167].

### piRNA in COPD

Chronic obstructive pulmonary disease (COPD) is an chronic inflammatory disease characterized by irreversible airway obstruction and abnormal inflammatory response in the lung [168]. Smoking is the most significant risk factor for COPD, involving various immune cells such as macrophages, neutrophils, dendritic cells, and CD8 + T cells, in its pathogenesis [169].

Sundar et al. analyzed small non-coding RNAs in plasma-derived extracellular vesicles through small RNA-seq and identified 34 distinct piRNAs across three groups: non-smokers, smokers and patients with COPD [170]. Pairwise comparisons revealed differentially expressed piRNAs: four piRNAs (piR-004153, piR-020813, piR-020450 and piR-016735) in smokers versus COPD patients, two piRNAs (piR-012753 and piR-020813) in non-smokers versus COPD patients, and three piRNAs (piR-004153, piR-020813 and piR-020450) in non-smokers versus smokers. Although the study primarily focused on miRNAs, these piRNAs may also serve as potential circulating biomarkers for COPD.

**Fig. 5:**
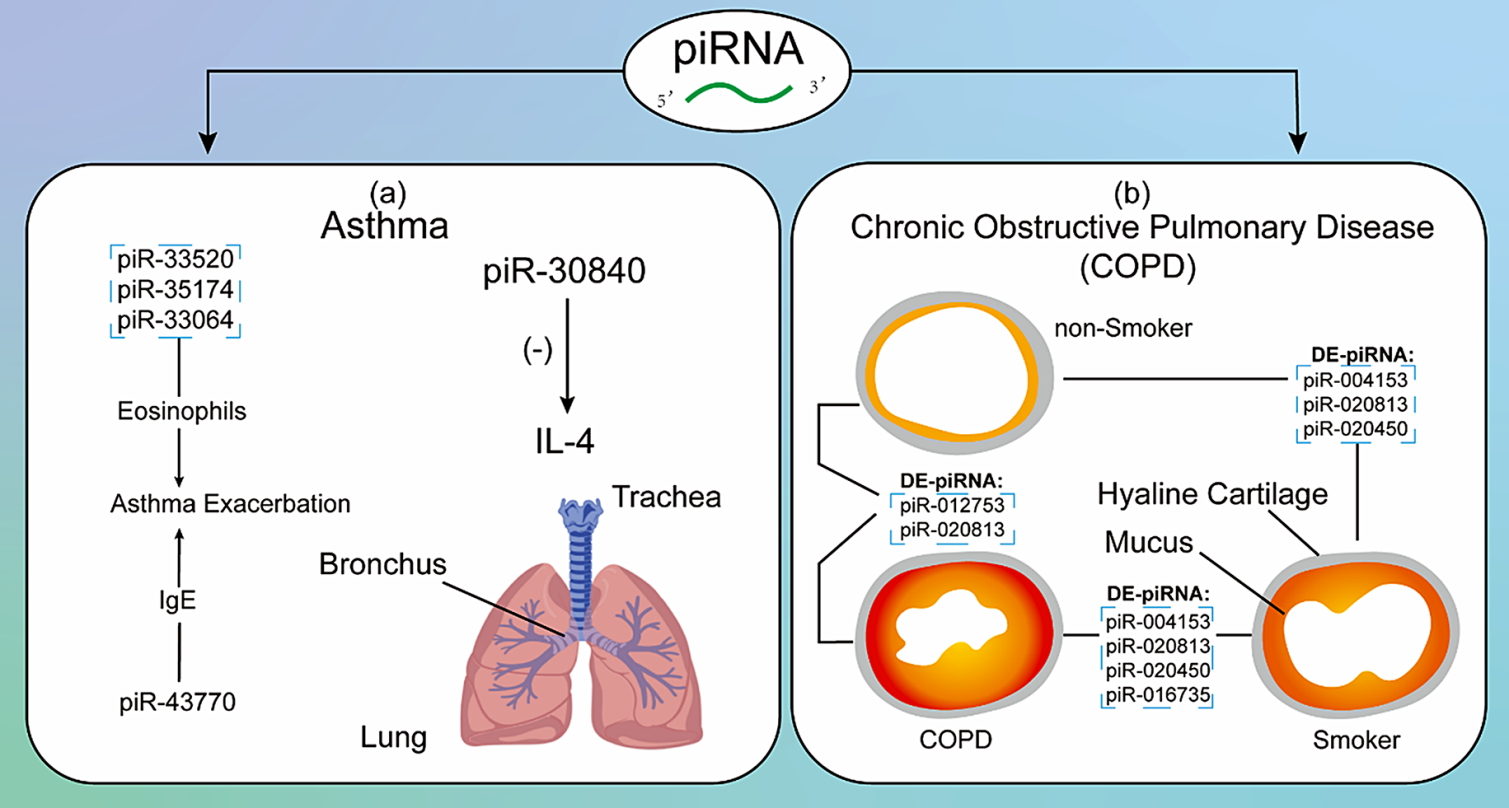
piRNA in Other Immune-Related Diseases. (**a**) piR-33,520, piR-35,174 and piR-33,064 are associated with eosinophils, while piR-43,770 is associated with serum total IgE. piRNA may influence asthma exacerbation through their effects on eosinophils and IgE. Additionally, piR-30,840 can downregulate IL-4 in CD4 + T cell. (**b**) Differentially expressed piRNAs between non-smokers, smokers and COPD patients. *Abbreviations*: DE: Differentially Expressed; IgE: Immunoglobulin E

## Conclusions

piRNAs were first discovered in germ cells, and numerous studies have since illuminated their pivotal role in transposon silencing [171]. Subsequently, piRNAs were also identified in somatic cells and even exosomes, drawing attention to their diverse functions beyond reproduction [18].

piRNAs have been extensively studied in various immune diseases. However, the majority of these investigations have focused on disease associations or biomarkers, leaving the underlying mechanisms of pathogenesis underexplored (as depicted in Table 3). For example, Han et al. revealed that piRNA-30,473 promotes DLBCL progression by modulating m6A RNA methylation [131]. Zhong et al. demonstrated that piR-30,840 downregulates IL-4 by binding to the intron region of pre-mRNA [76]. Jiang et al. discovered that most asthma-associated piRNAs overlap with enhancers, though the underlying mechanism remains elusive [167].

In some studies, tools designed for predicting miRNA target genes have been erroneously applied to piRNAs, raising concerns due to the significantly more intricate nature of seed matching during piRNA binding compared to miRNAs [140, 141, 148, 156, 159, 166]. Consequently, the interaction between piRNAs and their target genes should be experimentally validated using methods such as luciferase reporter assays and CLASH (cross-linking, ligation, and sequencing of hybrids) before being confidently addressed in research [71, 73].

Small RNA-seq is an effective method for discovering piRNAs, capable of identifying thousands of candidates in a single study [101, 108, 120, 121, 127, 145, 147, 155]. Therefore, employing a robust filtering strategy, which should encompass three key parts: quality control, alignment, and annotation, is crucial to eliminate noisy piRNAs [172]. The first step, quality control, involves removing adapters, trimming read sizes, and discarding low-quality reads to ensure qualified reads for downstream analysis. The alignment step maps the sequencing reads to the genome or piRNA database of the species. Configuring alignment parameters, such as allowing for mismatches and handling multiple alignments, is crucial for accurate results. Lastly, the annotation step significantly impacts the final outcome, as different piRNA databases contain varying piRNA records. Furthermore, small RNA-seq can detect numerous piRNA isoforms (isopiRs), yet the functional equivalence of these isopiRs to canonical piRNAs remains unclear [173].

In early piRNA databases, the criteria were not stringent, leading to the inclusion of some false positive piRNAs [174]. Additionally, some piRNAs may originate from other small non-coding RNAs [76]. Therefore, distinguishing genuine piRNAs from the vast output of high-throughput sequencing is crucial. Fortunately, current databases are placing great importance on experimental validation. For example, piRBase provides a gold standard piRNA dataset in its release v3.0 [9], and piRNA-IPdb collects piRNA sequences identified after immunoprecipitation with any of the PIWI proteins [89].

The field of piRNA research in immune diseases still holds vast untapped potential. Future studies should delve deeper into the mechanisms underlying piRNA’s role in immune diseases. For instance, elucidating the cytokines regulated by piRNAs and the potential pathways they participate in during disease pathogenesis are critical areas of investigation. piRNAs possess significant potential as diagnostic biomarkers, and targeting piRNAs with drugs or therapies offers promising therapeutic avenues.

**Table 3:** Summary of Key piRNAs in Immune diseases

piRNA^*^	Disease	Expression	Possible Mechanism or Biological Effect	Identification	Validation	Ref.
piR-33,044 (piR-16,659)	Rheumatoid arthritis	-	Increased by immune stimulus Poly(I: C).	RNA-Seq	RT-qPCR	[101, 102]
piR-35,411 (piR-hsa-27,620)		Up	-	RNA-Seq	RT-qPCR	[103]
piR-35,221 (piR-hsa-27,124)		Up	-	RNA-Seq	RT-qPCR	[103]
piR-35,866 (piR-020244)	Systemic Lupus Erythematosus	Down	Correlate with respiratory chain.	RNA-Seq	RT-qPCR	[108]
piR-35,463 (piR-019949)			-	RNA-Seq	RT-qPCR	
piR-61,723 (piR-018624)			-	RNA-Seq	RT-qPCR	
piR-53,563 (piR-16,771)	Multiple Sclerosis	-	Target MYC gene.	*In silico*	-	[20]
piR-54,038 (piR-17,232)		-		*In silico*	-	
piR-31,038 (piR-1177)		-		*In silico*	-	
piR-49,322 (hsa-piR-11,298)	Acquired Immune Deficiency Syndrome	Down	-	RNA-Seq	qPCR	[120]
piR-36,756 (hsa-piR-28,778)			-			
piR-33,432 (hsa-piR-23,408)			-			
piR-55,650 (hsa-piR-18,626)		Up	-			
piR-53,338 (hsa-piR-16,314)			-			
piR-38,716 (hsa-piR-30,738)			-			
piR-36,225	Chronic Lymphocytic Leukemia	Up	-	RNA-Seq	RT-qPCR	[127]
piR-30,799			-	RNA-Seq	RT-qPCR	[127]
piR-31,598 (piRNA-30,473)	Diffuse Large B-cell Lymphoma	Up	Mediate m6A methylation through regulating WTAP.	RNA-Seq	qPCR	[131]
(piR_017936)	Tuberculosis	-	Associated with the short-term interferon gamma release assay.	RNA-Seq	RT-qPCR	[155]
						[155]
piR-36,233	Herpes Simplex Virus	Up	Inhibit HSV-1 replication.	RNA-Seq	RT-qPCR	[141]
piR-36,041		Down	Promote HSV-1 replication.	RNA-Seq	RT-qPCR	[141]
piR-32,343 (piR-hsa-12,454)	Leprosy	Down	Regulate the GAS6 and IL6R pathways.	RNA-Seq	-	[159]
piR-57,947 (piR-hsa-21,131)						
piR-31,355 (piR-hsa-1580)						
piR-34,871 (piR-hsa-27,007)						
piR-36,499 (piR-hsa-28,634)						
piR-31,052 (piR-000753)	Brucellosis	Up	-	RNA-Seq	RT-qPCR	[161]
piR-31,925 (piR-001312)			-			
piR-33,161 (piR-016742)			-			
piR-30,840	Asthma	-	Downregulate IL-4 expression.	RNA-Seq	RT-qPCR	[76]
piR-43,770			Affect long-term asthma exacerbation through serum total IgE.	RNA-Seq	Replication in external cohort.	[167]
piR-33,520			Affect long-term asthma exacerbation through eosinophils.	RNA-Seq		
piR-35,174				RNA-Seq		
piR-33,064				RNA-Seq		
piR-43,772 (piR-004153)	Chronic Obstructive Pulmonary Disease	-	-	RNA-Seq	RT-qPCR	[170]
piR-36,712 (piR-020813)						
piR-36,170 (piR-020450)						
piR-33,151 (piR-016735)						
piR-54,381 (piR-012753)						

*The identifiers of piRNAs were mapped to the NCBI nucleotide database, with original names in brackets. However, piR_017936 failed in this mapping process

## Data Availability

No datasets were generated or analysed during the current study.

## Abbreviations

piRNA: PIWI-interacting RNA
PIWI: P-element-induced Wimpy Testis Protein
MiRNA: MicroRNA
siRNA: Small Interference RNA
Zuc: Zucchini
Aub: Aubergine
Ago3: Argonaute3
MHC: Major Histocompatibility Complex
RA: Rheumatoid Arthritis
OA: Osteoarthritis
SF: Synovial Fibroblasts
SLE: Systemic Lupus Erythematosus
LN: Lupus Nephritis
MS: Multiple Sclerosis
Hili: Human PIWI Protein
EV: Extracellular Vesicles
NSC: Neural Stem Cell
DE: Differentially Expressed
LTBI: Latent Tuberculosis Infection
CLL: Chronic Lymphocytic Leukemia
HIV: Human Immunodeficiency Virus
AIDS: Acquired Immune Deficiency Syndrome
DLBCL: Diffuse Large B-cell Lymphoma
TB: Tuberculosis
EVE: Endogenous Viral Elements
HSV: Herpes Simplex Virus
RSV: Respiratory Syncytial Virus
SARS-CoV-2: Severe Acute Respiratory Syndrome Coronavirus 2
M. leprae: Mycobacterium leprae
Ex/Mv: Exosomes/Microvesicles
HPV: Human Papilloma Virus
HNSCC: Head and Neck Squamous Cell Carcinoma
Th2: T Helper 2 Cells
IL4: Lnterleukin-4
IL6R: Interleukin 6 Receptor
CD4 + T: Cluster of Differentiation 4 Positive T cell
COPD: Chronic Obstructive Pulmonary Disease
